# Treatment of Neuralgia by Tobacco

**Published:** 1845-10

**Authors:** John Chippendale

**Affiliations:** London


*Treatment of Neuralgia by Tobacco.* By Mr. John Chippendale, London.—[In a former part of the Lancet from which we take the following extract, Mr. Chippendale suggests the use of tobacco in neuralgia; since then he has tried it successfully in two cases. Finding the infusion an inconvenient form for frictions, he ordered an extract to be made as follows:]

Take of the best shag tobacco, four ounces; distilled water, two pints; boil, and let them simmer for two or three hours, and strain; then wash the tobacco in two pints more of boiling distilled water, strain, and add it to the former liquor, and evaporate it to an extract. This operation was intrusted to the hands of Messrs. Baron and Harvey, the manufacturing druggists of Giltspur-street, and produced about an ounce and a half of the extract, possessing this very great advantage, as far as might regard private practice, that it was free from the disgusting smell belonging to tobacco. One part of this extract was now mixed with seven parts of simple cerate, to form an ointmdnt, which, for private practice, might be rendered more elegant, as the pharmacopolists say, by the addi tion of a little neroly, which I choose, as being as little stimulating as any essential oil of whieh I have any knowledge. Here, then, we have a narcotic ointment; for if the stimulating properties of the tobacco reside in the oil, it is to be presumed that has been all expelled in the manipulation. This ointment has been used for inunctions, in the quantity of half a drachm in some cases of neuralgia, night and morning, in others at night only, and has been attended with perfect success. The apothecary at the Farringdon Dispensary being applied to by a man with a bad toothache, advised the extraction of a tooth, but the patient not consenting to this, had some of this unguentum nicotianæ given to him for frictions, as a placebo, I believe, but it cured his roothache. I have also tried it as a local application, in psoriasis, with good effect; but on this it is not now my object to dwell.

It seems strange that Mr. Chippendale should have met with such success, considering that the same remedy failed in Dr. Allnatt's hands. Can it have been that the preparations of the herb used by the latter were of a stimulating character, while Mr. C's was a narcotic? It is always pleasant to be able to reconcile theory with fact, nothing being less satisfactory than empirical practice. Mr. Chippendale would account for the action of his strong preparation of tobacco, thus:—tobacco, it is notorious, deadens sensation; the agent is applied, so to speak, to a nerve itself: the first effect must be to deaden sensation in this nerve. The nerve is supplied with smaller nerves, nervi nervorum, or nervi vasorum, which are instrumental in the nutrition of that nerve; when these are dulled, their functions become diminished, whereby the nerve becomes atrophied, and the pain which was produced by pressure ceases. True, the process of nutrition may be again set up, and the neuralgia reinduced, but it is probable, that, by proper perseverance in the first instance, permanent atrophy might be produced.—[*Braithwaite's Retrospect.*

